# The Flavoring Agent Dihydrocoumarin Reverses Epigenetic Silencing and Inhibits Sirtuin Deacetylases

**DOI:** 10.1371/journal.pgen.0010077

**Published:** 2005-12-16

**Authors:** Andrew J Olaharski, Jasper Rine, Brett L Marshall, Joshua Babiarz, Luoping Zhang, Eric Verdin, Martyn T Smith

**Affiliations:** 1 School of Public Health, University of California Berkeley, Berkeley, California, United States of America; 2 Department of Molecular and Cellular Biology, University of California Berkeley, Berkeley, California, United States of America; 3 Gladstone Institute of Virology and Immunology, University of California San Francisco, San Francisco, California, United States of America; 4 Department of Medicine, University of California San Francisco, San Francisco, California, United States of America; Washington University in St. Louis, United States of America

## Abstract

Sirtuins are a family of phylogenetically conserved nicotinamide adenine dinucleotide-dependent deacetylases that have a firmly established role in aging. Using a simple *Saccharomyces cerevisiae* yeast heterochromatic derepression assay, we tested a number of environmental chemicals to address the possibility that humans are exposed to sirtuin inhibitors. Here we show that dihydrocoumarin (DHC), a compound found in *Melilotus officinalis* (sweet clover) that is commonly added to food and cosmetics, disrupted heterochromatic silencing and inhibited yeast Sir2p as well as human SIRT1 deacetylase activity. DHC exposure in the human TK6 lymphoblastoid cell line also caused concentration-dependent increases in p53 acetylation and cytotoxicity. Flow cytometric analysis to detect annexin V binding to phosphatidylserine demonstrated that DHC increased apoptosis more than 3-fold over controls. Thus, DHC inhibits both yeast Sir2p and human SIRT1 deacetylases and increases p53 acetylation and apoptosis, a phenotype associated with senescence and aging. These findings demonstrate that humans are potentially exposed to epigenetic toxicants that inhibit sirtuin deacetylases.

## Synopsis

The effects of chronic low-dose human exposure to environmental chemicals are difficult to study and poorly understood. Chemicals are routinely tested for the ability to induce DNA mutations, cause chromosome damage, or produce cell death, but are rarely tested for their ability to cause epigenetic changes, which can influence the behavior of a cell without directly changing the DNA sequence. Epigenetic changes have become the focus of intense research in an attempt to understand the mechanisms by which they function. The Sir2 family of deacetylases is one class of proteins that controls some epigenetic processes and, interestingly, has been implicated in extending the longevity of several organisms. Here the authors describe a novel assay based upon yeast Sir2p function to screen environmental chemicals for their ability to alter epigenetic silencing. From screening a relatively small number of agents, the authors found that dihydrocoumarin, a natural compound found in *Melilotus officinalis* (sweet clover) that is synthetically manufactured and frequently added to both food and cosmetics, disrupted epigenetic processes in the yeast *Saccharomyces cerevisiae.* Dihydrocoumarin also inhibited several human Sir2 family deacetylases (SIRT1 and SIRT2) and, when added to cells in culture, increased p53 tumor suppressor protein acetylation and caused elevated levels of apoptosis. The present study suggests that humans are exposed to a number of environmental chemicals that may be classified as epigenetic toxicants.

## Introduction

Members of the silent information regulator 2 *(SIR2)* family of genes encode highly conserved nicotinamide adenine dinucleotide (NAD^+^)-dependent deacetylases present in organisms from archaebacteria to eukaryotes [1]. In the yeast *Saccharomyces cerevisiae,* Sir2p is a histone deacetylase required for heterochromatic silencing at telomeres, ribosomal DNA, and mating type loci [2]. The sirtuin family of deacetylases has a firmly established role in aging [3]. Increased *SIR2* activity, mediated either by overexpression or through sirtuin-activating compounds, increases longevity in *S. cerevisiae* [4], *Drosophila melanogaster* [5], and *Caenorhabditis elegans* [5,6]. Conversely, *SIR2* deletion reduces life span in *S. cerevisiae* by 30% [7], and Sir2p inhibition by nicotinamide mimics this effect [8]. Seven apparent homologs of *SIR2* (SIRT1–7) exist in humans, with SIRT1 being the presumed Sir2p ortholog due to sequence similarity [1]. Although the human sirtuin deacetylases have a role in heterochromatin modification, they have mainly been identified to have nonhistone protein targets [9].

SIRT1 has been identified to deacetylate p53 [10] as well as a number of other proteins involved with the apoptotic response [10–13]. The p53 tumor suppressor protein is often referred to as the “guardian of the genome” due to its role in cell cycle arrest, senescence, and apoptosis [14]. Lysine acetylation (K320, K373, K382) increases p53 stability [15,16], leading to the transcriptional activation of DNA repair, cell cycle arrest, and proapoptotic genes. Because SIRT1-mediated p53 deacetylation reverses these effects, inhibition of this deacetylation step is hypothesized to promote p53 stability and increase apoptosis levels. Apoptosis and p53 stabilization accompany SIRT1 down-regulation [17], and SIRT1 inhibition by nicotinamide results in p53 hyperacetylation following DNA damage [10]. In addition, SIRT1^−/−^ cells have been identified to be more susceptible to killing by the genotoxic agents cisplatin and staurosporine [18], indicating that SIRT1 abrogation may enhance p53 function. SIRT1-deficient mice are observed to have developmental defects that are likely due to enhanced tumor suppression by a hyperacetylated and stable p53 [19]. p53 activity appears to control a fine balance between appropriate tumor suppression leading to cancer avoidance and stem cell depletion leading to tissue senescence [20,21]. SIRT1 balances these processes.

The discovery that resveratrol, a chemical found in red wine and other foods, increases life span in multiple organisms through a mechanism that may involve the activation of Sir2p [4,5] suggests that the diet and environment might also contain inhibitors of sirtuin enzymes. We devised a pair of haploid strains of the yeast *S. cerevisiae* that, if exposed to an agent that disrupts one kind of epigenetically heritable chromatin state, would mate and exhibit diploid growth on minimal medium. We tested over 100 chemicals to which humans are commonly exposed, including coumarins, bioflavonoids, benzene metabolites, essential oils, and arsenic, to address the possibility that these compounds could disrupt heterochromatin repression. Here we show that dihydrocoumarin (DHC), a compound found in *Melilotus officinalis* (sweet clover) that is widely used in the food and cosmetic industries [22,23], tested positive in the yeast mating assay and was identified to inhibit both Sir2p and SIRT1. Concentration-dependent increases in cytotoxicity, apoptosis, and p53 acetylation were observed following DHC exposure in the human TK6 lymphoblastoid cell line. These findings demonstrate that humans are exposed to epigenetic toxicants that inhibit sirtuin deacetylases.

## Results

### Development of a Screening Assay to Detect Environmental Chemicals That Disrupt Heterochromatin Silencing in *S. cerevisiae*

Haploid *S. cerevisiae* exists in two distinct mating types, **a** and **α**, controlled by a single locus on Chromosome III, *MAT.* Additionally, both **a** and **α** information are stored at the cryptic mating loci *HML* **α** and *HMR**a**.* These cryptic mating loci are used for mating-type interconversion and are silenced in part by Sir2p. Using a strain in which both *HML* and *HMR* contain **α** information and a recessive *mat**a**1–1* allele, disruption of any protein required for silencing, including Sir2p, either by chemical inhibition or mutation, would cause a phenotypic switch from an **a** to an **α** mating type. Mixing these cells with a strain of yeast that is *MAT**a** HMR**a** HML**a*** would result in mating only if heterochromatin silencing were inhibited. Mixing strains of these genotypes with complementary auxotrophic markers provided a simple test for agents that disrupt heterochromatic silencing, leading to mating and subsequent growth on minimal medium containing uracil. Using this technically simple indicator assay, we tested over 100 environmental chemicals to which humans are exposed (e.g., arsenic, benzene metabolites, coumarins, bioflavonoids, and essential oils, listed in Table S1, Supporting Information).

### DHC and *M. officinalis* Oil Extract Derepresses Heterochromatic Silencing in *S. cerevisiae*

**Figure 1:**
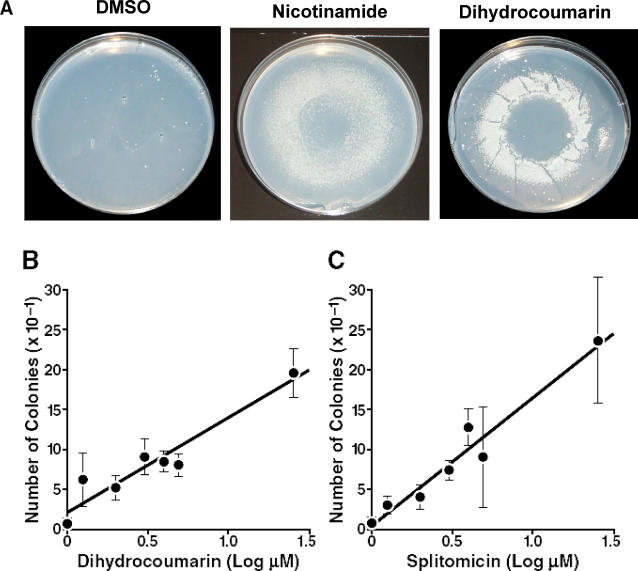
Effect of DHC on Heterochromatin Silencing in S. cerevisiae at the HMR or HML Loci (A) DMSO negative control, 1 M nicotinamide positive control, and 50 mM DHC. (B and C) DHC and splitomicin, respectively, cause dose-dependent increases in heterochromatin silencing and colony formation.

DHC (CAS# 119–84–6) from two independent sources derepressed heterochromatic gene silencing in the yeast *S. cerevisiae* mating assay (Figure 1). Oil extracts from *M. officinalis,* a common flowering plant that contains small amounts of DHC, also tested as a weak positive in the assay (data not shown). Colony growth following DHC exposure demonstrated a dose-response relationship at micromolar levels that was similar to the level of heterochromatin derepression observed with the established Sir2p inhibitor splitomicin (Figures 1B and 1C).

### DHC Is a Sir2p Inhibitor

**Figure 2:**
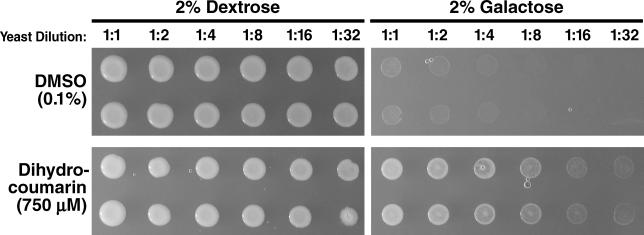
DHC Reverses the Lethal Phenotype of Galactose-Induced Plasmid *SIR2* Overexpression Two-fold yeast serial dilutions were used for this assay. Growth is observed on 0.1% DMSO and 750 μM DHC in dextrose medium. The lethal *SIR2* phenotype is observed on the 0.1% DMSO galactose medium but is partially reversed on the 750 μM DHC galactose medium.

The inhibition of heterochromatic silencing by DHC at levels similar to those observed with splitomicin suggested that it may be a Sir2p inhibitor; further experiments, however, were necessary to test whether Sir2p was the target. To this end, we employed a plasmid under a gal promoter that overexpresses *SIR2* and produces a lethal phenotype in the presence of galactose [24]. Addition of 750 μM DHC partially suppressed the Sir2p-induced lethality in galactose, but had no deleterious effect in glucose (Figure 2), thus pinpointing Sir2p as the target of DHC. Addition of a lower concentration of DHC (500 μM DHC) also reversed the lethal *SIR2* phenotype (data not shown). A recent report using a yeast telomeric reporter assay to analyze splitomicin analogs [25] support our finding that DHC is a yeast Sir2p inhibitor.

### DHC Inhibits Human SIRT1 and SIRT2

**Figure 3:**
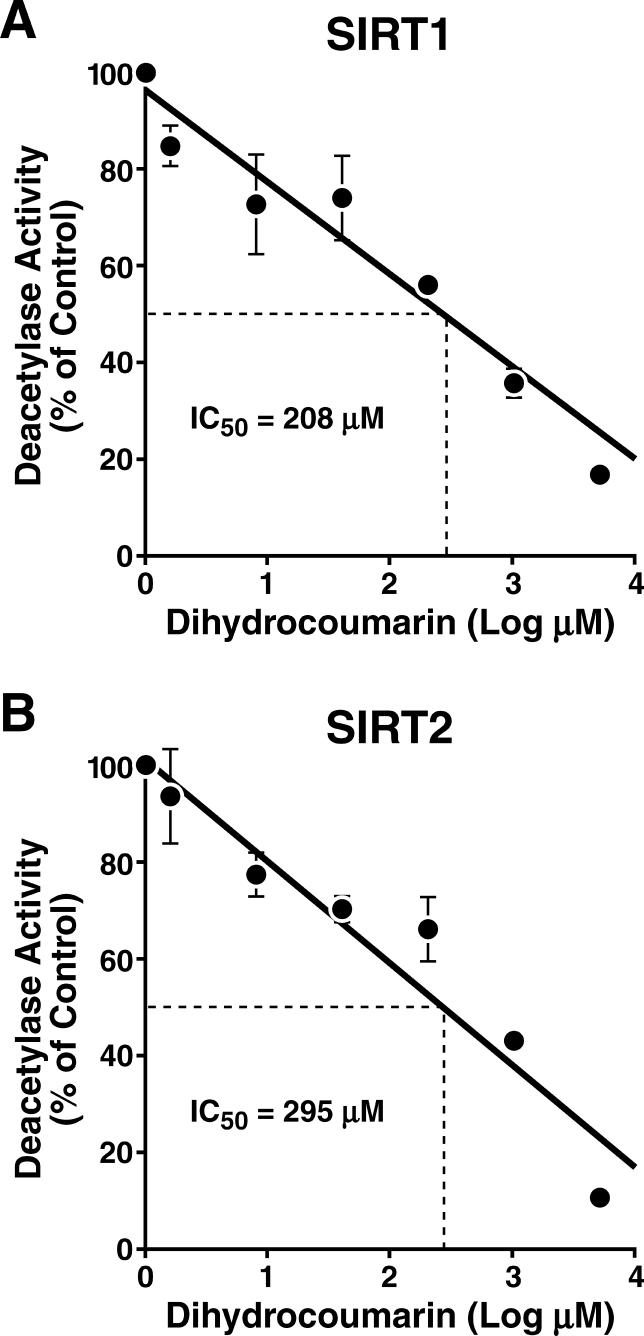
DHC Inhibits Human NAD^+^-Dependent Deacetylases In Vitro (A) Dose-dependent inhibition of SIRT1 (*y* = −19.98x + 96.431). (B) Dose-dependent inhibition of SIRT2 (*y* = −20.79x + 101.34). Averages ± standard error from two separate experiments are shown.

We tested whether DHC could inhibit the human NAD^+^-dependent deacetylase SIRT1 and observed that DHC induced a concentration-dependent inhibition of SIRT1 (IC_50_ of 208 μM) in an in vitro enzymatic assay (Figure 3A). A decrease in SIRT1 deacetylase activity was observed even at micromolar doses (85 ± 5.8 and 73 ± 13.7% activity at 1.6 μM and 8 μM, respectively). The microtubule SIRT2 [26] deacetylase was also inhibited with a similar dose dependency (Figure 3B).

### DHC Increases p53 Acetylation, Cytotoxicity, and Apoptosis Levels

**Figure 4:**
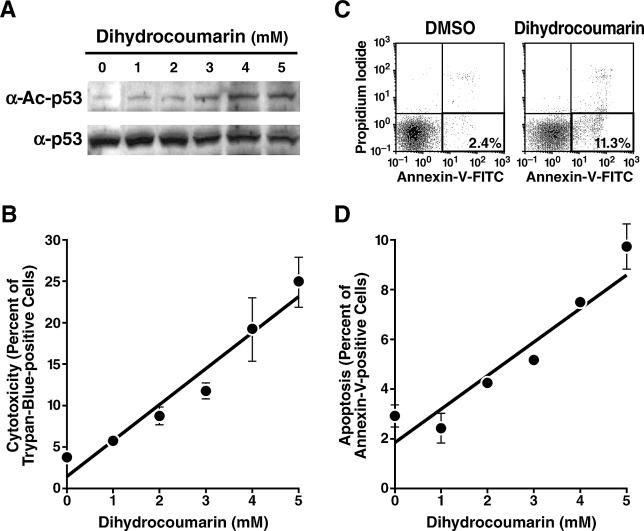
DHC Increases p53 Acetylation, Cytotoxicity, and Apoptosis in the TK6 Cell Line (A) DHC increases p53 lysine 373 and 382 acetylation in a dose-dependent manner in the TK6 cell line following a 24-h exposure period. The immunoblot is representative of three separate experiments. (B) DHC increases cytotoxicity in a dose-dependent manner following a 24-h exposure to DHC. The average ± standard error for each of three experiments is shown. (C) A 5-mM dose of DHC increases apoptosis at the 6-h time point in the TK6 cell line. A combination of annexin V fluorescein isothiocyanate and propidium iodide staining was used to discriminate among apoptotic (lower-right box) and necrotic (upper-right box) cells. Figures are representative of three separate experiments. (D) DHC increases apoptosis in a dose-dependent manner in the TK6 cell line at the 6-h time point. The average ± standard error for three experiments is shown.

Experiments with the human TK6 lymphoblastoid cell line further addressed DHC-mediated SIRT1 inhibition. Immunoblot analysis demonstrated that both p53 acetylation and cytotoxicity increased in a dose-dependent manner following DHC exposure (Figure 4A and 4B). Flow cytometric analysis measuring annexin V binding to phosphatidylserine demonstrated that apoptosis levels increased more than 3-fold following DHC exposure (Figure 4C and 4D).

## Discussion

Epigenetic drift is hypothesized to occur, in part, through exposure to environmental and dietary compounds that affect the processes responsible for the proper maintenance of the proteome and epigenome [27,28]. The recent discovery that significant differences in epigenetic markers exist in monozygotic twins who lived different lifestyles and spent little time together supports this environmentally driven epigenetic drift hypothesis [29]. Additionally, the finding that methoxychlor, a common DDT substitute used to control mosquitoes, and vinclozolin, a broadly used fungicide applied in the wine industry [30], caused transgenerational epigenetic effects up to the F4 generation [31] indicates that humans are likely exposed to epigenetic toxicants causing epigenetic drift on a regular basis. These are intriguing findings that raise the question of whether the diet and environment contain epigenetic toxicants that may also inhibit the sirtuin deacetylases.

We have designed a yeast mating assay to identify environmental chemicals that inhibit heterochromatic repression in *S. cerevisiae* to address the possibility that humans are exposed to epigenetic toxicants that inhibit sirtuin deacetylases. After screening more than 100 environmental chemicals to which humans are exposed, including coumarins, bioflavonoids, benzene metabolites, and arsenic, we identified that DHC disrupted heterochromatic repression (see Figure 1A). Further analyses demonstrated that DHC-mediated heterochromatic derepression caused yeast colony formation in a concentration-dependent manner that was similar to splitomicin (Figure 1B and 1C). Splitomicin is an established Sir2p inhibitor [32] with a structure similar to DHC, thus it was likely that DHC-mediated heterochromatic derepression in the mating assay was due to Sir2p inhibition. Further experiments with an overexpressing *SIR2*-induced death phenotype were conducted to identify if Sir2p was the target of DHC. DHC-mediated reversal of the *SIR2* overexpressed death phenotype (Figure 2) indicated that DHC is a Sir2p inhibitor and that this inhibition was responsible for the heterochromatic derepression observed in the mating assay. DHC thus joins a short list of established Sir2p inhibitors that includes nicotinamide, splitomicin, and sirtinol [33].

The identification that DHC is a Sir2p inhibitor is of particular interest because it is a natural component of *M. officinalis* and is synthetically manufactured for use as a common fragrance in perfumes, cosmetics, lotions, soaps, and as a flavoring agent in beverages and chewing gum [23,34]. DHC is present at concentrations above 100 ppm (670 μM) in gelatins, puddings, soft candy, frozen dairy products, and baked goods [22]. Due to the potential DHC exposure to humans, we tested whether it could also inhibit human sirtuin deacetylases and identified that DHC inhibited both SIRT1 and SIRT2 deacetylases in vitro (Figure 3A and 3B). We also measured SIRT3 deacetylase activity but did not identify it to be affected by DHC (data not shown). It is only recently that the cellular function of the human sirtuin deacetylases has begun to be revealed. Because there are at least seven human sirtuins [1], it is expected that the substrates and functions of each will vary. SIRT2 is a cytoplasmic protein identified to be a microtubule deacetylase [26], an observation that may help explain its requirement for proper progression through mitosis [35]. SIRT1 has been identified to deacetylate a variety of nuclear substrates [9], including several that are transcription factors regulating differentiation and development or proteins involved in the apoptotic response, including FOXO [12,36], ku70 [11], p53 [10], and MEF2 [37]. Although SIRT1 has been identified to have mostly nonhistone targets, there is growing evidence to indicate that SIRT1 interacts with a number of transcription factors with results that may mimic epigenetic modifications. How SIRT1 inhibitors can affect these processes is just beginning to be understood.

Because SIRT1 has been identified to deactivate p53 through deacetylation, experiments in the human TK6 lymphoblastoid cell line were conducted to identify if DHC could induce a phenotype consistent with SIRT1 inhibition and p53 activation. DHC was identified to increase p53 acetylation, cytotoxicity, and apoptosis levels in vitro (Figure 4). In addition, DHC enhanced cell killing to etoposide in both the TK6 human lymphoblastoid and human embryonic kidney 293 cell lines (data not shown), data compatible with the observation that SIRT1^−/−^ cells are more susceptible to killing by genotoxic agents [18]. Previous in vivo and in vitro studies have demonstrated that DHC is not a mutagen, clastogen, or aneugen [34], suggesting that it is unlikely that DHC is stabilizing p53 and increasing cytotoxicity and apoptosis through a genotoxic mechanism. Rather, these data are consistent with the phenotypes of SIRT1 compromised cells [10,17] and support the hypothesis that DHC-mediated SIRT1 inhibition enhances p53 stabilization and increases apoptosis. Increasing tumor suppression and the susceptibility of a cell to undergo apoptosis, however, cannot solely be viewed in a beneficial chemotherapeutic light, particularly in those cases where exposure to an apoptosis-inducing agent may be widespread. The deleterious effects of a hyperactive p53 have been demonstrated in a transgenic mouse model that displayed a shortened life span and a variety of early aging-associated phenotypes [38]. It is hypothesized that increased levels of apoptosis cause a rapid depletion of stem cells, leading to premature tissue senescence [20,21], possibly decreasing longevity. Increasing p53 tumor suppression through inhibition of SIRT1 may tip the cell-survival balance toward apoptosis in a cell that would otherwise not undergo programmed cell death. It is interesting to speculate whether sirtuin deacetylases, which have a role in regulating longevity in a variety of organisms, may also regulate longevity in mammals through the control of apoptosis. Further work is clearly needed to address the possibility that enhanced apoptosis through chemical-mediated inhibition of SIRT1 can increase tissue senescence and affect longevity.

Our finding that the common flavoring agent DHC inhibits sirtuin deacetylases linked to aging is potentially worrisome. SIRT1 inhibition may lead to epigenetic alterations as well as possible stem cell depletion and early tissue senescence due to increased levels of apoptosis. While no compounds to our knowledge are currently classified as “senescegens,” it is possible that a number of environmental chemicals can increase tissue senescence and aging. The present study demonstrates that a more extensive screening of dietary and environmental chemicals is needed to identify other epigenetic toxicants to which humans are commonly exposed.

## Materials and Methods

### Yeast mating assay.

JRY075 *(MATa, HMLa, HMRa, ade6, met, his4, leu2,* and *ura3)* and JRY80 *(mata1–1, HML*α, *HMR*α, *leu1, ura3,* and *ade2)* yeast strains [39] were combined in liquid rich-medium (YPD), and 50 μl of the mixture was spread onto YM plates containing 30 μg/ml uracil. Chemicals (Sigma, St. Louis, Missouri, United States; Indofine, Hillsborough, New Jersey, United States) were dissolved in DMSO and added to the YM uracil+ plates at 50 mM concentrations, whereas essential oils and plant oil extracts (Yerba Buena, Woodside, California, United States; Lhasa Kharnak, Berkeley, California, United States; and Nature's Apothecary, Bloomingdale, Illinois, United States) were added directly to the plates. Plates were incubated for at least 72 h at room temperature to allow for diploid growth on minimal medium containing uracil. Nicotinamide (1 M) and DMSO were used as the positive and solvent controls, respectively, for all experiments. Yeast colony DHC and splitomicin dose-response experiments were conducted to compare the ability of DHC to disrupt heterochromatin repression with an established Sir2p inhibitor. Varying doses (1 through 25 μM) of DHC and splitomicin were added directly to the YM and uracil medium.

### Inhibition of the *SIR2-*induced death phenotype.

The yeast strain YSH278 and the galactose-induced *SIR2* pSS3 plasmid were the kind gifts of Scott Holmes. The yeast strain YSH278 was transformed with the pSS3 plasmid containing galactose-inducible alleles and grown overnight in raffinose medium lacking leucine. Sets of 2-fold serial dilutions from these cultures were spotted onto YM glucose medium lacking leucine (−LEU) and on galactose medium lacking leucine (−LEU GAL). Both the dextrose and galactose plates contained 750 μM DHC or 0.1% DMSO as a solvent control. Plates were incubated for at least 72 h at room temperature.

### SIRT1 and SIRT2 deacetylase assays.

The histone deacetylation assay with recombinant SIRT1 and SIRT2 was performed, as described previously [40], using a [^3^H] acetylated histone H4 peptide.

### Cell culture and immunoblot analysis.

The human TK6 lymphoblastoid cell line (ATCC) was maintained in RPMI1640 medium (GIBCO, San Diego, California, United States) containing 10% FBS (Omega Scientific, Tarzana, California, United States) and 1% penicillin and streptomycin (Omega Scientific) under standard conditions. TK6 cells were exposed to 0.1% DMSO and 1, 2, 3, 4, and 5 mM DHC for 24 h at a concentration of 3.5 × 10^5^–5 × 10^5^. Total cell lysates were collected from 1.5 × 10^6^–2 × 10^6^ cells using 100 μl of radioimmunoprecipitation assay (RIPA) lysis buffer. Protein concentrations were determined by the Bradford assay (Bio-Rad, Hercules, California, United States). Equal protein amounts were resolved by PAGE, transferred onto nitrocellulose membranes, and imunoblotted for lysine 372 and 383 acetylated p53 (Upstate, Charlottesville, Virginia, United States). Protein was visualized using the ECL method per manufacturer's protocol (Amersham Biosciences, Little Chalfont, United Kingdom). Film (Kodak BioMax XAR; Eastman Kodak Corporation, Rochester, New York, United States) was exposed and developed using the Konica SRX-101 developer (Konica Minolta Medical Imaging USA, Wayne, New Jersey, United States).

### Cellular cytotoxicity and apoptosis assays.

Cellular cytotoxicity for the aforementioned TK6 DHC experiments was conducted using the Trypan blue exclusion assay. A minimum of 200 cells were analyzed for each dose and experiment. Annexin V apoptosis assays were performed in three additional experiments in which TK6 cells were exposed to 0.1% DMSO and 1, 2, 3, 4, and 5 mM DHC for 6 h. TK6 cells were prepared for flow cytometric annexin V analysis following the manufacturer's protocol (BD Biosciences Pharmingen, San Diego, California, United States), except that 5 μl of 1 mg/ml propidium iodide (PI) was used. At least 10,000 TK6 cells were analyzed on a Beckman Coulter EPICS XL-MCL flow cytometer (Fullerton, California, United States) using System II software for each experiment. Live cells were gated and analyzed for annexin V FITC and PI staining.

## Supporting Information

Table S1 List of Chemicals and Essential Oils Tested for Heterochromatic Repression in the Yeast Mating Assay (32 KB DOC)

## Abbreviations

DHC: dihydrocoumarin
NAD^+^: nicotinamide adenine dinucleotide
*SIR2*: silent information regulator 2
