# Supplementary material for: The Flavoring Agent Dihydrocoumarin Reverses Epigenetic Silencing and Inhibits Sirtuin Deacetylases
Source: PLoS Genet. 2005 Dec 16;1(6):e77. doi: 10.1371/journal.pgen.0010077 (PMC1315280; doi:10.1371/journal.pgen.0010077)
Supplement: Supplementary file 1 [file pgen.0010077.st001.doc]

| Supplemental Table 1: List of chemicals and essential oils tested for heterochromatic repression in the yeast mating assay | | | |
| --- | --- | --- | --- |
| 1,3-dimethyl-2-thiourea | chloramphenicol | lavender | salicylic acid |
| 1,4 benzoquinone | chloroquine | lemon grass | sassafras |
| 3,3'-methylene-bis(4-hydroxycoumarin | chlorotriphenyltin | marjoram | sodium arsenite |
| 3-iso butyl-1-methyl xanthine | citronella | mastic gum | sodium azide |
| 4-hydroxy-3-methoxyphenylacetic acid | clovebud | melilot | sodium butyrate |
| 4-methylaminophenol sulfate | colchicine | melphalan | sodium doexycholate monohydrate |
| 5-azacytidine | coumarin | mimosa | sodium orthovanadate |
| 8-quinolinol | cypress | mugwort | spearmint |
| acetic acid | dextran sulfate | myrrh gum | splitomicin |
| acridine orange | dihydrocoumarin | myrtle | sulfanilamide |
| allspice | dinitrochlorobenzene | nicotinic acid | taurine |
| almond | ethylmethane sulfonate | O-phenylenediamine dihydrochloride | tea tree |
| anise | etoposide | opopanax resin | tetramethyl benzidine |
| balsam | eucalyptus | palmarosa | tetramethylurea |
| basil | fennel seed | phenyl 1,4-benzoquinone | thimerosol |
| benzamide | fisetin | polythylenimine | thuja |
| benzene triol | formamide | potassium acetate | tobacco absolute |
| benzoin gum | frankincense | potassium dichromate | trichostatin a |
| bergamot | genistein | potassium thiocyanate | trimethylamine |
| cacodylic acid | ginger | p-phenylene-diamine dihydrochloride | valproic acid |
| cajeput | guaicol | psoralen | wintergreen |
| camphor | hydracortizone | pyridoxine | wormwood |
| catnip leaf | hydroquinone | quercetin | xanthine |
| celery seed | hyssop | reserpine | ylang ylang |
| chamomile | imidazole | resveratrol |  |
| chloral hydrate | juniper berry | rosewood |  |
